# The non-motile phenotype of *Salmonella hha ydgT *mutants is mediated through PefI-SrgD

**DOI:** 10.1186/1471-2180-11-141

**Published:** 2011-06-20

**Authors:** Lauren E Wallar, Andrew M Bysice, Brian K Coombes

**Affiliations:** 1 Department of Biochemistry and Biomedical Sciences, McMaster University, Hamilton, L8N 3Z5, ON, Canada; 2 Michael G. DeGroote Institute for Infectious Disease Research, Hamilton, L8N 3Z5, ON, Canada

## Abstract

**Background:**

Two ancestral nucleoid-associated proteins called Hha and YdgT contribute to the negative regulation of several virulence-associated genes in *Salmonella enterica *serovar Typhimurium. Our previous work showed that Hha and YdgT proteins are required for negative regulation of *Salmonella *Pathogenicity Island-2 and that *hha ydgT *double mutants are attenuated for murine infection. Interestingly, *hha ydgT *mutant bacteria exhibited a non-motile phenotype suggesting that Hha and YdgT have a role in flagellar regulation.

**Results:**

In this study we show that the non-motile phenotype of *hha ydgT *mutants is due to decreased levels of the master transcriptional regulator FlhD_4_C_2 _resulting in down-regulation of class II/III and class III flagellar promoters and lack of surface flagella on these cells. The horizontally acquired *pefI-srgD *region was found to be partially responsible for this phenotype since deletion of *pefI-srgD *in a *hha ydgT *deletion background resulted in transient restoration of class II/III and III transcription, expression of surface flagella, and motility in the quadruple mutant.

**Conclusion:**

These data extend our current understanding of the mechanisms through which Hha and YdgT regulate flagellar biosynthesis and further describe how *S*. Typhimurium has integrated horizontal gene acquisitions into ancestral regulatory networks.

## Background

The pathogenic nature of *Salmonella enterica *has been shaped by the horizontal acquisition of virulence determinants [1,2]. In *Salmonella enterica *serovar Typhimurium (*S*. Typhimurium), many virulence genes are organized in mobile elements such as pathogenicity islands, prophages, and the *Salmonella *virulence plasmid [3,4]. The increased pathogenic capacity conferred by such genes is dependent on their integration into ancestral regulatory networks of the cell, which can be accomplished by regulatory evolution following horizontal gene transfer [5].

The Hha/YmoA family of small nucleoid-associated proteins in *Enterobacteriaceae *[6] can participate in fine-tuning virulence gene expression in response to environmental cues [6,7]. For example, YmoA regulates expression of Yop proteins, YadA adhesin, Yst enterotoxin and invasin in *Yersinia enterocolitica *[7-9]. Hha negatively regulates the α-hemolysin genes *hlyCABD *in *Escherichia coli *[10], *hilA *encoded within *Salmonella *pathogenicity island 1 (SPI-1) in *S*. Typhimurium [11] and the locus of enterocyte effacement in enterohemorrhagic *E. coli *[12]. A third member, YdgT, similarly represses *hlyCABD *in *E. coli *[13]. We and others have shown that Hha and YdgT are repressors of the type III secretion system (T3SS) encoded in *Salmonella *Pathogenicity island 2 (SPI-2), where they provide an important negative regulatory input required for virulence [14-16]. Within their role as modulators of gene expression, Hha and YdgT repress other genes in horizontally acquired regions in *Salmonella *including the pathogenicity islands SPI-1 through SPI-5 and genes on the *Salmonella *virulence plasmid [16].

**Figure 1:**
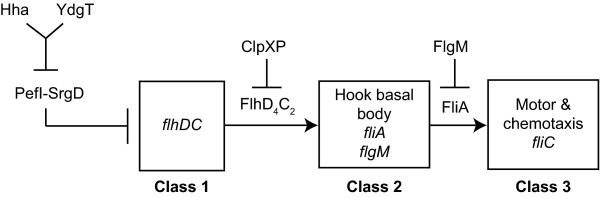
**Organization of the flagellar biosynthesis transcriptional hierarchy**. The early genes *flhDC *are transcribed from the class I promoter and encode the master transcriptional regulator FlhD_4_C_2 _which is able to bind within the class II promoter to activate transcription of the middle assembly genes in a σ^70^-dependent manner. The middle assembly genes encode the hook-basal body structure which spans the inner and outer membrane, the sigma factor FliA (σ^28^) and the anti-sigma factor FlgM. Once the hook-basal body is fully assembled, FlgM is exported through the hook-basal body allowing FliA to activate transcription of the late assembly genes from the class 3 promoter. Late assembly genes encode flagellin and proteins required for flagellar rotation and chemotaxis.

We and others have shown that *hha ydgT *mutants are non-motile [15,16], although the genetic basis linking the loss of Hha and YdgT to a non-motile phenotype was not known. Flagellar biosynthesis is an important virulence trait in enteric pathogens which can facilitate invasion of host intestinal epithelial cells [17]. Flagellar gene expression is governed by a three-tiered transcriptional hierarchy of early, middle, and late genes (Figure 1) [18]. The early genes *flhDC *encoding the master transcriptional regulator FlhD_4_C_2_, are at the top of the transcriptional hierarchy and are transcribed from the class I promoter [18]. FlhD_4_C_2 _in turn activates transcription of the middle genes encoding flagellar proteins comprising the hook-basal body, the alternative sigma factor FliA (σ^28^) and its anti-sigma factor FlgM [19]. Upon assembly of the hook-basal body, FlgM is secreted, releasing FliA to activate transcription of the late genes from the class III promoter [20,21]. The late genes encode flagellin, and motor and chemotaxis proteins [18]. Within the flagellar transcriptional hierarchy, multiple regulators acting at either class I or class II have been identified [21]. Recently, new regulatory genes (*pefI-srgD*) in the *pef *fimbrial operon on the *Salmonella *virulence plasmid were found to encode synergistic negative regulators of flagellar gene expression [22]. Interestingly, the *pefI-srgD *locus was upregulated ~7-fold in *hha ydgT *mutants [16] suggesting that Hha and YdgT might impinge on *pefI-srgD *for control of flagellar gene expression. We show here that deletion of *pefI-srgD *in a non-motile *hha ydgT *deletion mutant leads to a transient restoration of class II/III and class III gene expression that is sufficient for assembly of surface flagella and motility.

## Results

### Flagellar-based motility and surface flagellar expression is abolished in *hha ydgT *mutants

**Figure 2:**
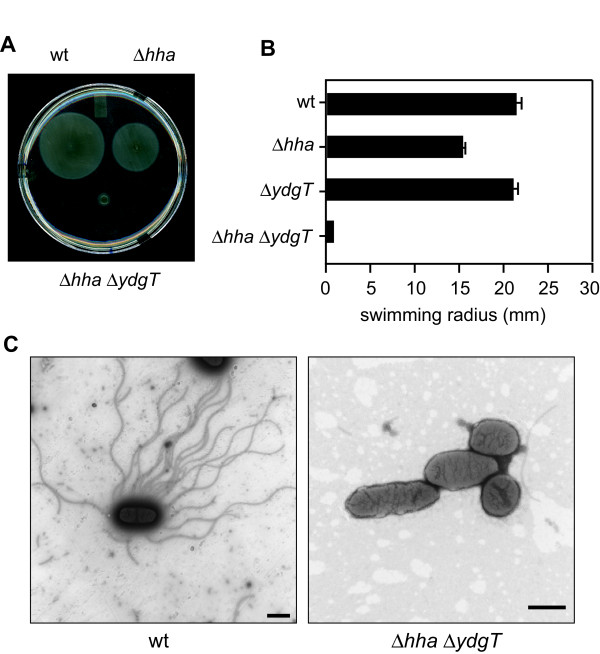
**Repression of flagellar biosynthesis and motility is dependent on the loss of Hha and YdgT**. (A) Wild type, Δ*hha*, Δ*ydgT *and Δ*hha *Δ*ydgT *were assessed for flagellar-based motility using a 0.25% soft agar motility assay in which 2 μL of overnight culture was inoculated into semi-solid agar and incubated at 37°C for 6 h. (B) The radius of the motility halo region was quantified after 6 h and is shown as means with standard errors. (C) Bacteria and surface flagella were negatively stained using a 0.1% uranyl acetate solution and visualized using scanning transmission electron microscopy. Data represents three independent experiments.

During our characterization of Hha and YdgT-mediated repression of SPI-2 genes, we noted that *hha ydgT *mutant bacteria settled to the bottom of standing culture tubes whereas wild type cultures remained turbid. Previous work indicated that *hha ydgT *mutants failed to swim on motility plates but the contribution of the individual genes to this phenotype was not known and the ability of these strains to make surface flagella was not tested [16]. To test the contribution of individual genes to this non-motile phenotype, we used a standard soft agar motility assay and confirmed that *hha ydgT *mutants were non-motile in accordance with previous data (Figure 2A). This phenotype required deletion of both *hha *and *ydgT *as single Δ*hha *or Δ*ydgT *mutants remained motile (Figure 2B). To determine if the motility defect observed in Δ*hha *Δ*ydgT *was due to a defect in flagellar rotation or a lack of flagellar production we stained bacteria and examined them using transmission electron microscopy to visualize surface flagella. We found that while wild type bacteria were highly flagellated, Δ*hha *Δ*ydgT *bacteria did not assemble flagella on their surface (Figure 2C).

### Transcriptional activity of class II/III and III promoters is decreased in a *hha ydgT *mutant

**Figure 3:**
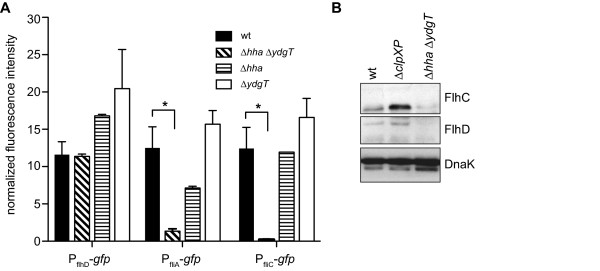
**Loss of Hha and YdgT disrupts flagellar biosynthesis at the level of Class II/III activation**. (A) Wild type and Δ*hha *Δ*ydgT *whole cell lysates were collected at OD_600 _~ 0.4-0.6 and levels of FlhC and FlhD were determined by Western blot analysis. DnaK was used as a loading control. (B) Promoter activity at Class I (*flhD*), II/III (*fliA*) and III (*fliC*) was determined in wild type, Δ*hha*, Δ*ydgT *and Δ*hha *Δ*ydgT *using GFP reporter plasmid constructs. Fluorescence intensity (501/511 nm) was measured after 6 h and normalized to OD_600 _(RLU/OD_600_). Data represents means and standard errors from three independent experiments.

Flagellar biosynthesis is organized into a transcriptional hierarchy of three distinct classes. To understand the non-flagellated phenotype in greater detail, we measured the activity of transcriptional reporters corresponding to each of the three promoter classes driving the expression of green fluorescent protein (GFP). While the transcriptional activity in single *hha *or *ydgT *mutants was not significantly different when compared to wild type, transcriptional reporters for the hybrid class II/III promoter (*fliA*) [23,24] and class III promoter (*fliC*) were significantly reduced in the *hha ydgT *double mutant compared to wild type cells (Figure 3A). Since *flhDC *promoter activity did not differ between wild type and the *hha ydgT *mutant, we tested whether the inhibition of class II/III and class III gene expression in Δ*hha *Δ*ydgT *involved an effect downstream of FlhD-FlhC protein production, since the FlhD_4_C_2 _complex is known to activate class II transcription. Using Western blot analysis with FlhC and FlhD-specific antisera, we observed a decrease in the levels of FlhC and FlhD in *hha ydgT *mutants compared to wild type (Figure 3B), which was consistent with the observed decrease in activity for FlhD_4_C_2 _target promoters. As a control we used a *clpXP *deletion mutant lacking the ClpXP protease that degrades the FlhD_4_C_2 _complex. As shown in Figure 3B, the levels of FlhC and FlhD were increased in Δ*clpXP *cells compared to wild type.

### Loss of the fimbrial regulators PefI-SrgD restores motility in a *hha ydgT *background

**Figure 4:**
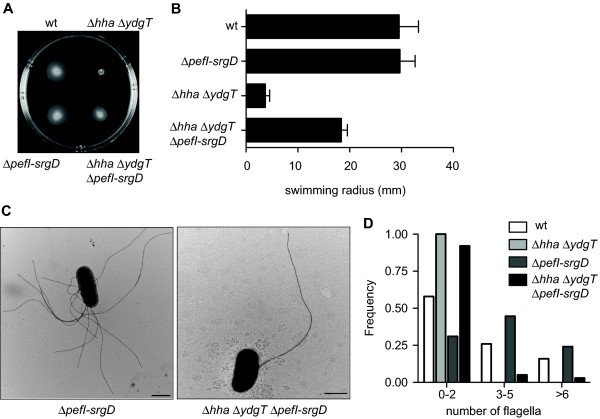
**Loss of PefI-SrgD restores flagellar biosynthesis and flagellar-based motility in Δ*hha *Δ*ydgT***. (A). Flagellar-based motility was determined in wild type, Δ*hha *Δ*ydgT*, Δ*pefI-srgD *and Δ*hha *Δ*ydgT *Δ*pefI-srgD *using a 0.25% soft agar motility assay. (B). The radius of the motility region was quantified after 6 h. (C). Bacteria and surface flagella were stained with 2% phosphotungstic acid and imaged using a transmission electron microscope. (D). Surface flagella were quantified for at least 100 bacteria cells for each strain. Motility assays are representative of three independent experiments and quantified as means and standard errors. TEM images are representative of two independent experiments.

We next wanted to identify potential negative regulators in Δ*hha *Δ*ydgT *that were acting to inhibit transcriptional regulation downstream of class I. Previous transcriptional profiling experiments showed that the *pefI-srgD *locus on the *Salmonella *virulence plasmid was upregulated ~7-fold following deletion of *hha ydgT *[16]. Subsequently, *pefI-srgD *genes were identified in a transposon mutagenesis screen as negative regulators of flagellar biosynthesis that worked in concert to inhibit motility [22]. Based on these data we hypothesized that the non-motile phenotype of *hha ydgT *mutants was mediated through its effect on *pefI-srgD*. If so, we reasoned that deletion of *pefI-srgD *in the *hha ydgT *mutant background would restore motility to this strain. We observed similar levels of motility (Figure 4A and Figure 4B) and surface flagella (Figure 4C and 4D) between wild type and Δ*pefI-srgD *bacteria, consistent with data from other groups [22]. However, as shown in Figure 4A, Figure 4B, and Figure 4C, deletion of *pefI-srgD *in the non-motile *hha ydgT *mutant restored surface flagella and motility to this strain. We noted that flagella distribution on the surface of Δ*hha *Δ*ydgT *Δ*pefI-srgD *quadruple mutants was less peritrichous and less abundant (Figure 4C and Figure 4D) than either wild type or Δ*pefI-srgD *suggesting that other regulators in addition to PefI-SrgD might be involved in regulating motility through the Hha and YdgT nucleoid-like proteins.

### Class II/III and class III promoters are transiently activated upon loss of PefI-SrgD in Δ*hha *Δ*ydgT *bacteria

**Figure 5:**
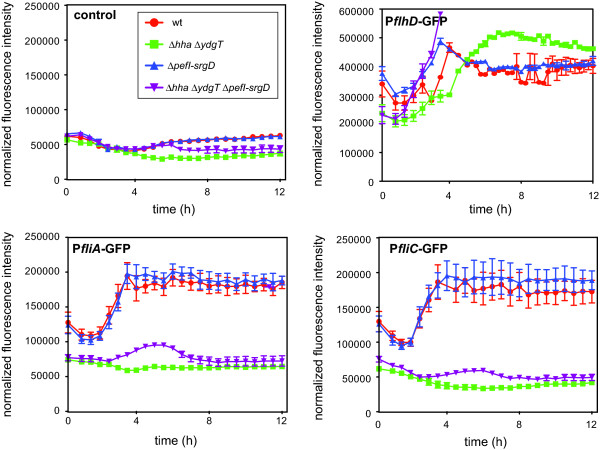
**Loss of PefI-SrgD induces transient but sufficient Class II/III and III activation to restore flagellar biosynthesis in Δ*hha *Δ*ydgT***. Promoter activity at each transcriptional class in wild type, Δ*hha *Δ*ydgT*, Δ*pefI-srgD *and Δ*hha *Δ*ydgT *Δ*pefI-srgD *was measured as fluorescence intensity using plasmid-based GFP reporters. A promoterless GFP reporter construct was used as a negative control (first panel). Fluorescence intensity (485/525 nm) and OD_600 _was measured at 15 min intervals over 19 h. Data represents fluorescence intensity normalized to OD_600 _(RLU/OD_600_). GFP transcriptional reporter assay data is representative of three independent experiments and quantified as means and standard errors (at the 3 h time point for P*flhD*, *P *< 0.05 for wt vs. Δ *pefI-srgD *and wt vs. Δ*hha *Δ*ydgT *Δ*pefI-srgD; *ANOVA, Newman-Keuls multiple comparison test). After 3-5 hours, P*flhD-gfp *activity in the quadruple mutant reached the maximum detection limit of the fluorescence reader. Data is shown for 12 hours rather than for 19 hours for the remaining flagellar reporters as there was no change in the fluorescence levels from 12-19 hours.

In transcriptional reporter experiments we were not able to detect class II/III or class III flagellar promoter activity in *hha ydgT *mutant bacteria despite similar class I gene expression levels relative to wild type. To determine if the restoration of motility in the Δ*hha *Δ*ydgT *Δ*pefI-srgD *mutant correlated with an increase in class II/III and class III promoter activity, we introduced the *gfp *transcriptional reporters into the *pefI-srgD *double mutant and the *hha ydgT pefI-srgD *quadruple mutant and measured promoter activity over time. Consistent with its role as a negative regulator of class I gene expression [22], P*flhD-gfp *activity was elevated in strains deleted for *pefI-srgD *compared to wild type, including the *hha ydgT pefI-srgD *mutant which showed the highest level of *flhD *promoter activity at ~3 h. In line with this, the quadruple mutant had a gain of transcriptional activity at class II/III and class III promoters that was apparent between 4-6 h (Figure 5). Although the level of reporter activity for the hybrid class II/III and class III reporters did not reach that of wild type cells, it was sufficient to restore the expression of surface flagella as shown by transmission electron microscopy, and to restore motility levels to ~80% of wild type.

## Discussion

We have shown that Hha and YdgT positively regulate flagellar biosynthesis through their influence on the horizontally acquired flagellar regulators PefI-SrgD. The ability of Hha and YdgT to act as positive regulators is manifested only in the presence of both proteins, as single deletions of *hha *and *ydgT *had no apparent effect on flagellar biosynthesis. A similar phenomenon has been observed in the regulation of α-haemolysin production in *E. coli*. Loss of both Hha and YdgT was required to dramatically de-repress α-haemolysin production which correlated with the ability of YdgT to attenuate the *hha *mutant phenotype [13]. Similarly, Hha and YdgT may be able to compensate for any effect on flagellar biosynthesis in the single deletion mutants making it difficult to discern their individual roles in flagellar biosynthesis regulation.

PefI-SrgD were recently identified as negative regulators of flagellar gene expression as they inhibit class I activation at the top of the flagellar biosynthesis transcriptional hierarchy [22]. PefI-SrgD is located within the *pef *fimbrial operon on the *Salmonella *virulence plasmid and PefI acts to regulate *pef *fimbriae expression [25,26]. Pef fimbriae are involved in bacterial adherence and fluid accumulation in the murine small intestine [27]. Phylogenetic data indicates that *S*. Typhimurium acquired *pef *as part of the serovar-specific virulence plasmid [28] which carries variable genetic elements required for virulence, fimbriae synthesis, plasmid transmission, innate immune resistance and antibiotic resistance [29,30].

The dual regulatory action of PefI-SrgD on both *pef *and flagellar promoters is similar to that seen for the regulation of fimbriae and flagella in other pathogens. PapX in uropathogenic *E. coli *acts to reciprocally regulate the expression of type 1 fimbriae and flagella during urinary tract infection [31]. MrpJ in *Proteus mirabilis*, an opportunistic urinary tract pathogen, activates MR/P fimbrial production while simultaneously repressing flagellar expression [32]. FimZ in *S*. Typhimurium coordinates reciprocal expression of type 1 fimbriae and flagella [33]. The existence of regulatory proteins able to dually control fimbriae and flagella production thus appears as an important evolutionary mechanism allowing tight modulation of adherence or motility phenotypes.

Although deletion of *pefI-srgD *in *hha ydgT *mutants de-represses the motility defect by re-establishing expression of surface flagella, it does not fully reconstitute class II/III and class III promoter activity to wild type levels suggesting the existence of other negative flagellar regulators. The protease ClpXP has been shown to degrade FlhD_4_C_2 _in *S*. Typhimurium [34], which may represent another negative regulatory mechanism in *hha ydgT *mutants.

The role of PefI-SrgD in the negative regulation of flagellar biosynthesis exemplifies the evolutionary significance of integrating horizontally acquired regulators into ancestral networks. For example, in *S*. Typhimurium, the horizontally acquired two-component regulatory system SsrA-SsrB regulates ancestral genes throughout the *Salmonella *genome [5,35]. In extraintestinal pathogenic *E. coli*, the horizontally acquired regulator Hfp interacts with the nucleoid-associated protein H-NS to regulate ancestral genes [36]. In *Shigella flexneri*, Sfh is located on a horizontally acquired virulence plasmid and regulates the expression of the ancestral proteins H-NS and StpA [37]. Thus, horizontal acquisition of regulatory proteins can have a significant impact on ancestral gene expression often by interacting with other regulatory pathways.

## Conclusions

We have shown that the non-motile phenotype of Δ*hha *Δ*ydgT *requires the loss of both Hha and YdgT and that this phenotype is partially mediated through PefI-SrgD. These data contribute to our understanding of Hha-and YdgT-dependent flagellar biosynthesis regulation and demonstrate the integration of the horizontally acquired regulators PefI-SrgD into the flagellar biosynthesis network.

## Methods

### Bacterial Strains and Mutant Construction

Bacteria were propagated in Luria-Bertani (LB) broth at 37°C with aeration unless otherwise indicated. Marked, in-frame deletions of *clpXP *and *pefI-srgD *were made in *Salmonella enterica *serovar Typhimurium SL1344 using the λ Red Recombinase method [38]. Generation of Δ*hha *Δ*ydgT *was described previously [15] and this strain was used to generate mutants incorporating the *pefI-srgD *deletion using the primers pefI-srgDF: GTG ATA CTT ATC CGG CCT CCG GTC CGC ATT CCA GGC CGG CCA TAT GAA TAT CCT CCT TAG and pefI-srgDR ATT CCG GTT TAT GAG TGA ATC CAT TGT TAC AAA AAT TAT TGT GTA GGC TGG AGC TGC TTC.

### Soft Agar Motility Assay

Two μl of overnight culture was inoculated into 0.25% LB Agar motility plates with antibiotic and incubated at 37°C for 6 h.

### Immunoblotting

Wild type and mutant strains were cultured until the optical density at 600 nm (OD_600_) reached ~ 0.4-0.6. Whole cell lysates were collected and probed using anti-FlhC (1:5000), anti-FlhD (1:2500) and anti-DnaK (1:5000, Stressgen) antibodies. DnaK served as a loading control.

### Transmission Electron Microscopy

Flagella were negatively stained using two different methods. In the first method, cells were cultured for 3-6 h. A carbon-stabilized Formvar support on 200-mesh copper TEM grid was floated for 30 seconds on a drop of culture, washed three times with water and stained for 10 seconds using 0.1% uranyl acetate. The second method involved staining copper grid-immobilized cells for 60 seconds with 2% phosphotungstic acid. Images were obtained using a JEOL-1200EX transmission electron microscope at the McMaster University Electron Microscopy Facility. For quantification, overnight cultures were diluted 1:50 or 1:100 in LB media with antibiotic and grown for at least 3 hours under static conditions. Flagella were stained as described above and quantified for at least 100 cells.

### Transcriptional Reporter Assays

Wild type cells and the various mutants under study were transformed with the plasmid-based green fluorescent protein (GFP) reporter constructs pP*_flhD_-GFP*, pP*_fliA_-GFP*, pP*_fliC_-GFP *and pP*_less_-GFP *published previously [39]. For reporter experiments, strains were either sub-cultured into culture tubes and propagated for 6 h at which point fluorescence intensity and OD_600 _were measured or strains were sub-cultured into 96-well plates in M9 media containing 0.1% casamino acids and antibiotic and grown with shaking at 37°C at 1080 cycles per minute. Fluorescence intensity and OD_600 _were measured at 15 minute intervals for 19 h using a Synergy 2 Multi-Mode Microplate Reader (Fisher Scientific Co).

## Authors' contributions

LEW, AB and BKC conceived and designed experiments and analyzed data; LEW, AB and BKC performed experiments; LEW and BKC wrote the paper. All authors read and approved the final manuscript.

## References

[B1] Porwollik S McClelland M Lateral gene transfer in *Salmonella* Microbes Infect 2003 5 11 977 989 10.1016/S1286-4579(03)00186-2 12941390

[B2] Dobrindt U Hochhut B Hentschel U Hacker J Genomic islands in pathogenic and environmental microorganisms Nat Rev Microbiol 2004 2 5 414 424 10.1038/nrmicro884 15100694

[B3] Baumler AJ Tsolis RM Ficht TA Adams LG Evolution of host adaptation in *Salmonella enterica* Infect Immun 1998 66 10 4579 4587 9746553 10.1128/iai.66.10.4579-4587.1998 PMC108564

[B4] Brussow H Canchaya C Hardt WD Phages and the evolution of bacterial pathogens: from genomic rearrangements to lysogenic conversion Microbiol Mol Biol Rev 2004 68 3 560 602 10.1128/MMBR.68.3.560-602.2004 15353570 PMC515249

[B5] Osborne SE Walthers D Tomljenovic AM Mulder DT Silphaduang U Duong N Lowden MJ Wickham ME Waller RF Kenney LJ Pathogenic adaptation of intracellular bacteria by rewiring a *cis*-regulatory input function Proc Natl Acad Sci USA 2009 106 10 3982 3987 10.1073/pnas.0811669106 19234126 PMC2645909

[B6] Madrid C Nieto JM Juarez A Role of the Hha/YmoA family of proteins in the thermoregulation of the expression of virulence factors Int J Med Microbiol 2002 291 6-7 425 432 11890540 10.1078/1438-4221-00149

[B7] Mikulskis AV Cornelis GR A new class of proteins regulating gene expression in enterobacteria Mol Microbiol 1994 11 1 77 86 10.1111/j.1365-2958.1994.tb00291.x 8145648

[B8] Cornelis GR Sluiters C Delor I Geib D Kaniga K Lambert de Rouvroit C Sory MP Vanooteghem JC Michiels T *ymoA*, a *Yersinia enterocolitica *chromosomal gene modulating the expression of virulence functions Mol Microbiol 1991 5 5 1023 1034 10.1111/j.1365-2958.1991.tb01875.x 1956283

[B9] Ellison DW Young B Nelson K Miller VL YmoA negatively regulates expression of invasin from *Yersinia enterocolitica* J Bacteriol 2003 185 24 7153 7159 10.1128/JB.185.24.7153-7159.2003 14645275 PMC296258

[B10] Nieto JM Carmona M Bolland S Jubete Y de la Cruz F Juarez A The *hha *gene modulates haemolysin expression in *Escherichia coli* Mol Microbiol 1991 5 5 1285 1293 10.1111/j.1365-2958.1991.tb01902.x 1956303

[B11] Fahlen TF Wilson RL Boddicker JD Jones BD Hha is a negative modulator of transcription of *hilA*, the *Salmonella enterica *serovar Typhimurium invasion gene transcriptional activator J Bacteriol 2001 183 22 6620 6629 10.1128/JB.183.22.6620-6629.2001 11673432 PMC95493

[B12] Sharma VK Carlson SA Casey TA Hyperadherence of an *hha *mutant of *Escherichia coli *O157:H7 is correlated with enhanced expression of LEE-encoded adherence genes FEMS Microbiol Lett 2005 243 1 189 196 10.1016/j.femsle.2004.12.003 15668018

[B13] Paytubi S Madrid C Forns N Nieto JM Balsalobre C Uhlin BE Juarez A YdgT, the Hha paralogue in *Escherichia coli*, forms heteromeric complexes with H-NS and StpA Mol Microbiol 2004 54 1 251 263 10.1111/j.1365-2958.2004.04268.x 15458420

[B14] Coombes BK Wickham ME Lowden MJ Brown NF Finlay BB Negative regulation of *Salmonella *pathogenicity island 2 is required for contextual control of virulence during typhoid Proc Natl Acad Sci USA 2005 102 48 17460 17465 10.1073/pnas.0505401102 16301528 PMC1297660

[B15] Silphaduang U Mascarenhas M Karmali M Coombes BK Repression of intracellular virulence factors in *Salmonella *by the Hha and YdgT nucleoid-associated proteins J Bacteriol 2007 189 9 3669 3673 10.1128/JB.00002-07 17307861 PMC1855891

[B16] Vivero A Banos RC Mariscotti JF Oliveros JC Garcia-del Portillo F Juarez A Madrid C Modulation of horizontally acquired genes by the Hha-YdgT proteins in *Salmonella enterica *serovar Typhimurium J Bacteriol 2008 190 3 1152 1156 10.1128/JB.01206-07 18039769 PMC2223552

[B17] Knodler LA Vallance BA Celli J Winfree S Hansen B Montero M Steele-Mortimer O Dissemination of invasive *Salmonella *via bacterial-induced extrusion of mucosal epithelia Proc Natl Acad Sci USA 2010 107 41 17733 17738 10.1073/pnas.1006098107 20876119 PMC2955089

[B18] Chilcott GS Hughes KT Coupling of flagellar gene expression to flagellar assembly in *Salmonella enterica *serovar typhimurium and *Escherichia coli* Microbiol Mol Biol Rev 2000 64 4 694 708 10.1128/MMBR.64.4.694-708.2000 11104815 PMC99010

[B19] Wozniak CE Hughes KT Genetic dissection of the consensus sequence for the class 2 and class 3 flagellar promoters J Mol Biol 2008 379 5 936 952 10.1016/j.jmb.2008.04.043 18486950 PMC2488150

[B20] Aldridge PD Karlinsey JE Aldridge C Birchall C Thompson D Yagasaki J Hughes KT The flagellar-specific transcription factor, sigma28, is the Type III secretion chaperone for the flagellar-specific anti-sigma28 factor FlgM Genes Dev 2006 20 16 2315 2326 10.1101/gad.380406 16912280 PMC1553213

[B21] Chevance FF Hughes KT Coordinating assembly of a bacterial macromolecular machine Nat Rev Microbiol 2008 6 6 455 465 10.1038/nrmicro1887 18483484 PMC5963726

[B22] Wozniak CE Lee C Hughes KT T-POP array identifies EcnR and PefI-SrgD as novel regulators of flagellar gene expression J Bacteriol 2009 191 5 1498 1508 10.1128/JB.01177-08 19114490 PMC2648201

[B23] Kalir S McClure J Pabbaraju K Southward C Ronen M Leibler S Surette MG Alon U Ordering genes in a flagella pathway by analysis of expression kinetics from living bacteria Science 2001 292 5524 2080 2083 10.1126/science.1058758 11408658

[B24] Brown JD Saini S Aldridge C Herbert J Rao CV Aldridge PD The rate of protein secretion dictates the temporal dynamics of flagellar gene expression Mol Microbiol 2008 70 4 924 937 18811728 10.1111/j.1365-2958.2008.06455.x

[B25] Friedrich MJ Kinsey NE Vila J Kadner RJ Nucleotide sequence of a 13.9 kb segment of the 90 kb virulence plasmid of *Salmonella typhimurium*: the presence of fimbrial biosynthetic genes Mol Microbiol 1993 8 3 543 558 10.1111/j.1365-2958.1993.tb01599.x 8100983

[B26] Nicholson B Low D DNA methylation-dependent regulation of *pef *expression in *Salmonella typhimurium* Mol Microbiol 2000 35 4 728 742 10.1046/j.1365-2958.2000.01743.x 10692151

[B27] Baumler AJ Tsolis RM Bowe FA Kusters JG Hoffmann S Heffron F The pef fimbrial operon of *Salmonella typhimurium *mediates adhesion to murine small intestine and is necessary for fluid accumulation in the infant mouse Infect Immun 1996 64 1 61 68 8557375 10.1128/iai.64.1.61-68.1996 PMC173728

[B28] Baumler AJ Gilde AJ Tsolis RM van der Velden AW Ahmer BM Heffron F Contribution of horizontal gene transfer and deletion events to development of distinctive patterns of fimbrial operons during evolution of *Salmonella *serotypes J Bacteriol 1997 179 2 317 322 8990281 10.1128/jb.179.2.317-322.1997 PMC178699

[B29] Chu C Chiu CH Evolution of the virulence plasmids of non-typhoid *Salmonella *and its association with antimicrobial resistance Microbes Infect 2006 8 7 1931 1936 10.1016/j.micinf.2005.12.026 16713725

[B30] Rotger R Casadesus J The virulence plasmids of *Salmonella* Int Microbiol 1999 2 3 177 184 10943411

[B31] Simms AN Mobley HL PapX, a P fimbrial operon-encoded inhibitor of motility in uropathogenic *Escherichia coli* Infect Immun 2008 76 11 4833 4841 10.1128/IAI.00630-08 18710869 PMC2573324

[B32] Li X Rasko DA Lockatell CV Johnson DE Mobley HL Repression of bacterial motility by a novel fimbrial gene product EMBO J 2001 20 17 4854 4862 10.1093/emboj/20.17.4854 11532949 PMC125589

[B33] Clegg S Hughes KT FimZ is a molecular link between sticking and swimming in *Salmonella enterica *serovar Typhimurium J Bacteriol 2002 184 4 1209 1213 10.1128/jb.184.4.1209-1213.2002 11807085 PMC134799

[B34] Tomoyasu T Takaya A Isogai E Yamamoto T Turnover of FlhD and FlhC, master regulator proteins for *Salmonella *flagellum biogenesis, by the ATP-dependent ClpXP protease Mol Microbiol 2003 48 2 443 452 10.1046/j.1365-2958.2003.03437.x 12675803

[B35] Tomljenovic-Berube AM Mulder DT Whiteside MD Brinkman FS Coombes BK Identification of the regulatory logic controlling *Salmonella *pathoadaptation by the SsrA-SsrB two-component system PLoS Genet 2010 6 3 e1000875 10.1371/journal.pgen.1000875 20300643 PMC2837388

[B36] Muller CM Schneider G Dobrindt U Emody L Hacker J Uhlin BE Differential effects and interactions of endogenous and horizontally acquired H-NS-like proteins in pathogenic *Escherichia coli* Mol Microbiol 2010 75 2 280 293 10.1111/j.1365-2958.2009.06995.x 19968792 PMC2814080

[B37] Deighan P Beloin C Dorman CJ Three-way interactions among the Sfh, StpA and H-NS nucleoid-structuring proteins of *Shigella flexneri *2a strain 2457T Mol Microbiol 2003 48 5 1401 1416 10.1046/j.1365-2958.2003.03515.x 12787365

[B38] Datsenko KA Wanner BL One-step inactivation of chromosomal genes in *Escherichia coli *K-12 using PCR products Proc Natl Acad Sci USA 2000 97 12 6640 6645 10.1073/pnas.120163297 10829079 PMC18686

[B39] Cummings LA Wilkerson WD Bergsbaken T Cookson BT In vivo, *fliC *expression by *Salmonella enterica *serovar Typhimurium is heterogeneous, regulated by ClpX, and anatomically restricted Mol Microbiol 2006 61 3 795 809 10.1111/j.1365-2958.2006.05271.x 16803592

